# Weighted Lomax distribution

**DOI:** 10.1186/s40064-016-3489-2

**Published:** 2016-10-24

**Authors:** N. M. Kilany

**Affiliations:** Faculty of Science, Menoufia University, Shebin El-Kom, Egypt

**Keywords:** Lomax distribution, Weighted distribution, Reliability measures, Method of moments estimation, Maximum likelihood estimation method

## Abstract

The Lomax distribution (Pareto Type-II) is widely applicable in reliability and life testing problems in engineering as well as in survival analysis as an alternative distribution. In this paper, Weighted Lomax distribution is proposed and studied. The density function and its behavior, moments, hazard and survival functions, mean residual life and reversed failure rate, extreme values distributions and order statistics are derived and studied. The parameters of this distribution are estimated by the method of moments and the maximum likelihood estimation method and the observed information matrix is derived. Moreover, simulation schemes are derived. Finally, an application of the model to a real data set is presented and compared with some other well-known distributions.

## Introduction

Weighted distribution theory gives a unified approach to dealing with model specification and data interpretation problems. Weighted distributions occur frequently in studies related to reliability, survival analysis, analysis of family data, biomedicine, ecology and several other areas, see Stene (1981) and Oluyede and George (2002). Many authors have presented important results on weighted distributions, Rao (1965) introduced a unified concept of weighted distribution and identified various sampling situations that can modeled by weighted distributions. These situations occur when the recorded observations can not be considered as a random sample from the original distributions. This mean that sometimes it is not possible to work with a truly random sample from population of interest. Zelen (1974) introduced weighted distribution to represent what broadly perceived as a length-biased sampling. Patil and Ord (1976) studied a size biased sampling and related invariant weighted distributions. Statistical applications of weighted distributions related to human population and ecology can be found in Patil and Rao (1978). Gupta and Tripathi (1996) studied the weighted version of the bivariate logarithmic series distribution, which has applications in many fields such as: ecology, social and behavioral sciences.

To present the concept of a weighted distribution, suppose that *X* is a nonnegative random variable with its probability density function (p.d.f.) *f*(*x*), then the p.d.f. of the weight random variable *X*_*w*_ is given by

1 $$f_{w} \left( x \right) = \frac{w(x)f(x)}{E(w(x))},\quad x \ge 0$$

where $w\left( x \right)$ is a nonnegative weight function and $E\left( {w\left( x \right)} \right) = \int \nolimits_{0}^{\infty } w\left( x \right)f\left( x \right)dx , 0 < E\left( {w\left( x \right)} \right) < \infty$. The random variable *X*_*w*_ is called the weight version of *X* and its distribution is related to *X* and is called the weighted distribution with weight function $w\left( x \right)$. Note that, the weight function *w*(*x*) in Eq. (1) gave different practical examples: such as; when $w\left( x \right) = x^{\beta } ,\beta > 0$, then the resulting distribution is called a size biased version of *X* and the p.d.f. of a size random variable *X*_*s*_ is defined as

2 $$f_{s} \left( x \right) = \frac{{x^{\beta } f\left( x \right)}}{{E\left( {x^{\beta } } \right)}}, \quad x \ge 0,\quad \alpha > 0$$

In Eq. (2) when $\beta$ = 1, then the weight function $w\left( x \right) = x$ and the resulting distribution is called a length-biased distribution and the p.d.f. of a length biased random variable *X*_*L*_ is taken the following form:

$$f_{L} \left( x \right) = \frac{xf\left( x \right)}{E\left( x \right)}, \quad x \ge 0,\quad \alpha > 0$$

where $E\left( X \right) = \mu$ is the mean of the original distribution [to know more details about different forms of a weight function, see Rao (1985) and Hewa (2011)]. When an investigator records an observation by nature according to certain stochastic model, the recorded observation will not have the original distribution unless every observation is given an equal chance of being recorded. For example, suppose that the original observation $x_{0}$ comes from a distribution with p.d.f $f_{0} \left( {x_{0} ;\theta_{1} } \right)$, where $\theta_{1}$ is a parameter vector, and that observation *x* is recording according to re-weighted by weighted function $w\left( {x;\theta_{2} } \right) > 0, \theta_{2}$ is a new parameter vector, then *x* comes from a distribution with p.d.f.

$$f\left( x \right) = Aw\left( {x;\theta_{2} } \right)f_{0} \left( {x_{0} ;\theta_{1} } \right)$$

where A is a normalized constant.

The main aim of this paper is to provide another extension of the Lomax distribution. So, the Weighted-Lomax (“WLx” for short) distribution is proposed to offer a more flexible model for modelling data in several areas such as lifetime analysis, engineering and biomedical sciences. The objectives of the research are to study some structural properties of the proposed distribution. Lomax (1987) proposed Lomax distribution (Pareto Type-II distribution), and used it for the analysis of the business failure lifetime data. Lomax distribution often used in business, economics, and actuarial modeling. It is essentially a Pareto distribution that has been shifted so that its support begins at zero. A random variable *X* is said to be distributed as Lomax with two parameters *α* (shape parameter) and *λ* (scale parameter) if it has p.d.f.,

3 $$f_{L} \left( {x;\alpha ,\lambda } \right) = \frac{{\alpha \lambda^{\alpha } }}{{\left( {y + \lambda } \right)^{\alpha + 1} }},\quad x \ge 0,\quad \alpha , \quad \lambda > 0$$

The corresponding cumulative distribution function (c.d.f.) given by

4 $$F_{L} \left( {x;\alpha ,\lambda } \right) = 1 - \left[ {1 + \left( {\frac{x}{\lambda }} \right)} \right]^{ - \alpha }$$

The trend of parameter(s) induction to the baseline distribution has received increased attention in recent years to explore properties and for efficient estimation of the parameters. In the literature, some extensions of Lomax distribution are available such as the exponentiated Lomax by Abdul-Moniem and Abdel-Hameed (2012), Marshall–Olkin extended-Lomax by Ghitany et al. (2007), Gupta et al. (2010), Beta-Lomax (BL), Kumaraswamy Lomax, McDonald-Lomax by Lemonte and Cordeiro (2013), Gamma-Lomax by Cordeiro et al. (2015), the generalized transmuted Lomax by Nofal et al. (2016) and the transmuted Weibull Lomax by Afify et al. (2015).

In this paper, the WLx distribution is proposed with p.d.f.

$$f\left( x \right) = Ax^{\beta - 1} f_{L} \left( {x;\alpha ,\lambda } \right)$$

where A is a normalized constant and $f_{L} \left( {x;\alpha ,\lambda } \right)$ is the p.d.f. of Lomax distribution. Using Eq. (3), the WLx considered in this paper has p.d.f.

5 $$f\left( x \right) = \frac{{\Gamma \left( {\alpha + 1} \right)\lambda^{1 + \alpha - \beta } }}{{\varGamma \left( {1 + \alpha - \beta } \right)\varGamma \left( \beta \right)}}\left( {\frac{{x^{\beta - 1} }}{{\left( {x + \lambda } \right)^{\alpha + 1} }}} \right) ,\quad x \ge 0,\quad \lambda > 0, \quad \alpha > 0,\quad 0 < \beta < \alpha + 1$$

where $\Gamma \left( \cdot \right)$ is the complete gamma function. Note that, when $\beta = 1$, the weighted Lomax distribution reduces to the Lomax distribution. The corresponding c.d.f. is

6 $$F\left( x \right) = \frac{{\Gamma \left( {\alpha + 1} \right)\lambda^{ - \beta } x^{\beta } \times _{2} F_{1} \left( {\alpha + 1,\beta ,\beta + 1; - \frac{x}{\lambda }} \right)}}{{\beta\Gamma \left( \beta \right)\Gamma \left( {1 + \alpha - \beta } \right)}}$$

where _2_F_1_(*a*, *b*, *c*; *z*) is the hypergeometric function.

This paper is organized as follows: In “Distributional properties” section the distributional properties of the proposed distribution are derived and studied. Section “Parameter estimation” discusses the estimation problem using the method of moment and the maximum likelihood estimates of the model parameters. The order statistics and the limiting distribution of the extreme values are derived in “Order statistics and extreme values” section. Section “Simulation study” includes the simulation study for WLx distribution. Finally, a real life application is illustrated in “Conclusion” section.

## Distributional properties

### Shapes of probability density function

The behavior of p.d.f. of the WLx distribution $f\left( x \right)$ at *x* = 0 and $x = \infty$, respectively, is given by

$$f\left( 0 \right) = \left\{ {\begin{array}{*{20}l} {\infty , } \hfill & \quad{if\;\beta < 1} \hfill \\ {\frac{\alpha }{\lambda },} \hfill & \quad{if\;\beta = 1,\quad \quad f(\infty ) = 0} \hfill \\ {0,} \hfill & \quad{if\;\beta > 1} \hfill \\ \end{array} } \right.$$

The following theorem describes the shape of p.d.f. of the WLx distribution.

#### **Theorem 1**

*The p.d.f.* $f\left( x \right)$ *of the WLx distribution is*

(i) *Decreasing if* $$\{ 0\left\langle {\beta \le 1, \alpha } \right\rangle 0 , \lambda > 0\}$$.
(ii) *Unimodal if* $\left\{ {1\left\langle {\beta \left\langle {\alpha + 1, \alpha } \right\rangle 0 , \lambda } \right\rangle 0} \right\}$.

#### *Proof*

 The first derivative of $f\left( x \right)$ is given by

$$f^{'} \left( x \right) = \frac{g\left( x \right)}{{x\left( {x + \lambda } \right)}}f\left( x \right)$$

where

$$g\left( x \right) = \left( { - 2 - \alpha + \beta } \right)x + \left( {\beta - 1} \right)\lambda$$

(i) If $\beta = 1,$ then $g\left( x \right) = - \left( {\alpha + 1} \right)x < 0$. Hence $f^{\prime}\left( x \right) < 0$ which implies that $f\left( x \right)$ is decreasing. If $\beta < 1$, then $g\left( x \right) < 0$ for all $\alpha + 1\left\langle {\beta , \lambda } \right\rangle 0$. Hence $f\left( x \right)$ is decreasing.
(ii) If $\left\{ {1\left\langle {\beta \left\langle {\alpha + 1, \alpha } \right\rangle 0 , \lambda } \right\rangle 0} \right\}$, $f^{\prime}\left( x \right) = 0$ *iff* $g\left( x \right) = 0$ which occurs at the point
  $$x_{0} = \frac{{\left( {\beta - 1} \right)\lambda }}{2 + \alpha - \beta }$$

Since

$$f^{''} \left( x \right) = \frac{{ - \left( {2 + \alpha - \beta } \right)}}{{x_{0} \left( {x_{0} + \lambda } \right)}}f\left( {x_{0} } \right) < 0,$$

**Fig. 1:**
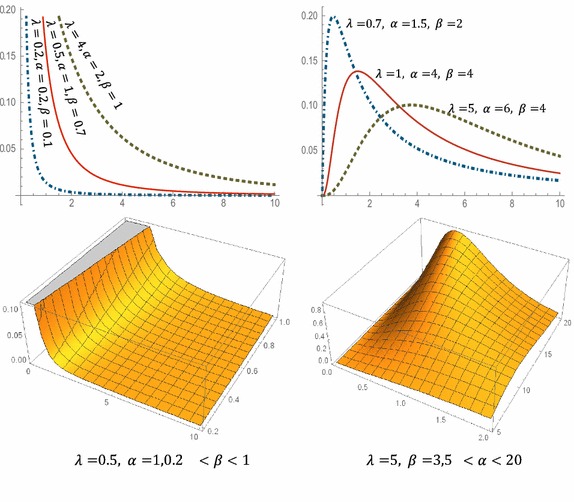
Plots of the WLx p.d.f. for some parameter values

Hence $f\left( x \right)$ has a local maximum at $x_{0}$. The behavior of WLx distribution density can be illustrated as in the Fig. 1.

### Hazard rate function

From (5) and (6), the survival and the hazard (or failure) rate functions are obtained by

$$\begin{aligned} S\left( x \right) = 1 - F\left( x \right) = 1 - \frac{{\Gamma \left( {\alpha + 1} \right)\lambda^{ - \beta } x^{\beta } \times\, _{2} F_{1} \left( {\alpha + 1,\beta ,\beta + 1; - \frac{x}{\lambda }} \right)}}{{\beta\Gamma \left( \beta \right)\Gamma \left( {1 + \alpha - \beta } \right)}} \hfill \\ h\left( x \right) = \frac{f\left( x \right)}{S\left( x \right)} = \frac{{\Gamma \left( {\alpha + 1} \right)\beta \lambda^{\alpha + 1} x^{\beta - 1} \left( {x + \lambda } \right)^{ - \alpha - 1} }}{{\beta \lambda^{\beta }\Gamma \left( {1 + \alpha - \beta } \right)\Gamma \left( \beta \right) - x^{\beta }\Gamma \left( {1 + \alpha } \right) \times _{2} F_{1} \left( {\alpha + 1,\beta ,\beta + 1; - \frac{x}{\lambda }} \right)}} \hfill \\ \end{aligned}$$

According to Glaser (1980), in order to study the behavior of $h\left( x \right)$, the behavior of $\eta \left( x \right)$ is examined where $\eta \left( x \right) = - \frac{d}{dx}\ln f\left( x \right)$. The following theorem shows the shapes of the hazard rate function of the WLx distribution.

#### **Theorem 2**

*Hazard rate function of the WLx distribution is*

(i) *Decreasing if* $$\{ 0\left\langle {\beta \le 1, \alpha } \right\rangle 0 , \lambda > 0\}$$.
(ii) *Upside-down shape if* $\left\{ {1\left\langle {\beta \left\langle {\alpha + 1, \alpha } \right\rangle 0 , \lambda } \right\rangle 0} \right\}$.

#### *Proof*

 Since

$$\eta \left( x \right) = - \frac{d}{dx}\ln f\left( x \right) = \frac{1 - \beta }{x} + \frac{\alpha + 1}{x + \lambda }$$

It follows that

$$\eta^{{\prime }} \left( x \right) = - \frac{1 - \beta }{{x^{2} }} - \frac{\alpha + 1}{{\left( {x + \lambda } \right)^{2} }},\quad \eta^{{\prime \prime }} \left( x \right) = \frac{{2\left( {1 - \beta } \right)}}{{x^{3} }} + \frac{{2\left( {\alpha + 1} \right)}}{{\left( {x + \lambda } \right)^{3} }}$$

(i) For $0 < \beta \le 1$, $\eta^{\prime}\left( x \right) < 0$ for all $\alpha + 1\left\langle {\beta , \lambda } \right\rangle 0$, i.e. $\eta \left( x \right)$ is decreasing. Hence, $h\left( x \right)$ is also decreasing.
(ii) For $1 < \beta < \alpha + 1$, $\eta^{\prime}\left( x \right) = 0$ occurs at two points

$$x_{1} = \frac{{ - \lambda \left( {\beta - 1} \right) + \lambda \sqrt {\left( {\beta - 1} \right)\left( {\alpha + 1} \right)} }}{ - 2 - \alpha + \beta },\quad x_{2} = \frac{{ - \lambda \left( {\beta - 1} \right) - \lambda \sqrt {\left( {\beta - 1} \right)\left( {\alpha + 1} \right)} }}{ - 2 - \alpha + \beta }$$

Since,

$$\eta^{{\prime \prime }} \left( {x_{2} } \right) = - \frac{{2\left( {\alpha + 1} \right)\left( {2 + \alpha - \beta } \right)^{4} \left( {\beta - 1} \right)\left( {\sqrt {\beta - 1} - \sqrt {\alpha + 1} } \right)^{2} }}{{\lambda^{3} \left( {\sqrt {\left( {\beta - 1} \right)\left( {\alpha + 1} \right)} \left( {\sqrt {\beta - 1} + \sqrt {\alpha + 1} } \right)^{2} } \right)^{3} }} < 0,$$

**Fig. 2:**
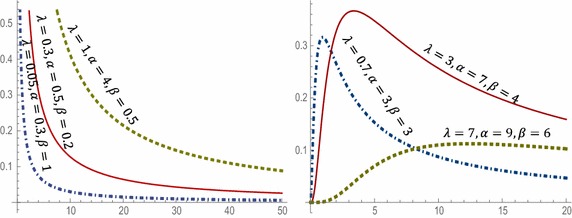
Plots of the Hazard function of WLx distribution

Then $\eta \left( x \right)$ has a local minimum at $x_{2}$ and therefore the hazard function has a local minimum at $x_{2}$ then upside-down shaped. Plots of the WLx hazard function at different parameter values are displayed in Fig. 2.

### Mean residual life function and reversed failure rate

In life testing situations, the remaining lifetimes of a unit of age $x \ge 0$ until the time of failure is known as the residual life. In other words, if $X$ is the life of a unit, then the conditional random variable $\left[ {\left( {X - x} \right)/X > x} \right]$ is called the residual life ($RL$). Gupta and Gupta (1983) showed that the mean residual life function and also the ratio of any two consecutive moments of residual life characterize the distribution. The mean residual life (MRL) function of WLx distribution is given by:

$$\begin{aligned} MRL & = \mu_{1} \left( x \right) = E\left( {X - x/X > x} \right) = \frac{1}{S\left( x \right)}\int \nolimits_{x}^{\infty } yf\left( y \right)dy - x \\ & = - x + \frac{{\beta \left( { - \lambda } \right)^{ - \alpha + \beta } \lambda^{\alpha + 1} {\text{B}}\left( { - \frac{\lambda }{x};\alpha - \beta , - \alpha } \right)\Gamma \left( {\alpha + 1} \right)}}{{\beta \lambda^{\beta }\Gamma \left( {1 + \alpha - \beta } \right)\Gamma \left( \beta \right) - x^{\beta }\Gamma \left( {\alpha + 1} \right) \times _{2} F_{1} \left( {\alpha + 1,\beta ,\beta + 1; - \frac{x}{\lambda }} \right)}} \\ \end{aligned}$$

where ${\text{B}}\left( {x;a,b} \right)$ is the incomplete beta function. The following two Lemmas are useful to determining the shape of mean residual life function *µ*(*x*).

#### **Lemma 2**

 (Bryson and Siddique 1969) *Let X be a non-negative continuous random variable with hazard rate function h(x) and mean residual life function µ(x). If h(x) is increasing (decreasing), then µ(x) is increasing (decreasing).*

#### **Lemma 3**

 (Gupta and Akman 1995) *Let X be a non-negative continuous random variable with p.d.f. f(x), hazard rate function h(x) and mean residual life function µ(x). If h(x) has bathtub (upside-down bathtub) shaped and f(0)µ(0)* *>* *1(=* *1), then µ(x)has upside-down bathtub (bathtub) shape.*

Using Lemmas 2 and 3, the following theorem shows the shape of the mean residual life function *µ*(*x*) of the WLx distribution.

#### **Theorem 3**

*The mean residual life function µ(x) of the WLx distribution is increasing (bathtub shaped) if* $0 < \beta \le 1$ ($1 < \beta < \alpha + 1$) *for all* $\alpha > 0 , \lambda > 0$.

#### *Proof*

**Fig. 3:**
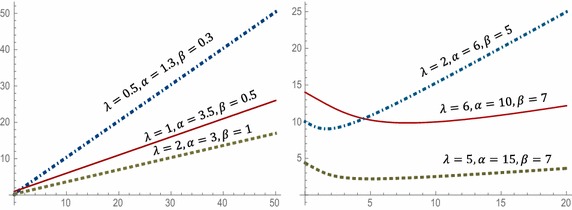
Plots of the mean residual life of WLx distribution

 Since *h*(*x*) is decreasing for $0 < \beta \le 1$, then *µ*(*x*) is increasing. Moreover, since *h*(*x*) is upside-down shaped for $1 < \beta < \alpha + 1$ and *f*(0)*µ*(0) $\le$ 0, *µ*(*x*) is bathtub shaped. Figure 3 illustrates the plot of mean residual life function of WLx distribution at different parameter values.

The reversed residual life can be defined as the conditional random variable $\left[ {x - X/X \le x} \right]$ which denotes the time elapsed from the failure of a component given that its life is less than or equal to *x*, $x \ge 0$. This random variable is called also the inactivity time or time since failure.

In addition, in reliability, it is well known that the mean reversed residual life and ratio of two consecutive moments of reversed residual life characterize the distribution uniquely; for more details see Kundu and Nanda (2010) and Nanda et al. (2003). The reversed failure for WLx Distribution is derived as follows:

$$Rh\left( x \right) = \frac{f\left( x \right)}{F\left( x \right)} = \frac{{\beta \lambda^{1 + \alpha } \left( {x + \lambda } \right)^{ - 1 - \alpha } }}{{x \times _{2} F_{1} \left( {\alpha + 1,\beta ,\beta + 1; - \frac{x}{\lambda }} \right)}}$$

The rth moment of the reversed residual life is obtained by:

$$m_{r} \left( x \right) = E\left( {\left( {x - X} \right)^{r} \text{ / }X \le x} \right) = \frac{1}{F\left( x \right)}\int \nolimits_{0}^{x} r\left( {x - t} \right)^{r - 1} F\left( t \right)dt$$

Thus the mean of the reversed residual life is:

$$m_{1} \left( x \right) = \frac{{x \times\, _{2} F_{1} \left( {\alpha + 1,\beta ,\beta + 1; - \frac{x}{\lambda }} \right)}}{{\left( {\beta + 1} \right) \times\, _{2} F_{1} \left( {\alpha + 1,\beta ,\beta + 1; - \frac{x}{\lambda }} \right)}}$$

The second moment of the reversed residual life is:

$$m_{2} \left( x \right) = \frac{{\lambda^{2} {\text{B}}\left( { - \frac{x}{\lambda };2 + \beta , - \alpha } \right)}}{{{\text{B}}\left( { - \frac{x}{\lambda };\beta , - \alpha } \right)}} + x^{2} \left( { - 1 + \frac{{2 \times\, _{2} \tilde{F}_{1} \left( {1 + \alpha ,\beta ,2 + \beta , - \frac{x}{\lambda }} \right)}}{{ _{2} \tilde{F}_{1} \left( {1 + \alpha ,\beta ,1 + \beta , - \frac{x}{\lambda }} \right)}}} \right)$$

where, $_{2} \tilde{F}_{1} \left( {a,b,c;z} \right) = \frac{{ _{2} F_{1} \left( {a,b,c;z} \right)}}{\varGamma \left( c \right)}$ is the regularized hypergeometric function.

### Moments and associated measures

The first four moments about the origin of WLx distribution

7 $$\mu^{\prime}_{1} = \frac{\beta \lambda }{\alpha - \beta }$$

8 $$\mu^{\prime}_{2} = \frac{{\beta \left( {\beta + 1} \right)\lambda^{2} }}{{\left( { - 1 + \alpha - \beta } \right)\left( {\alpha - \beta } \right)}}$$

9 $$\mu^{\prime}_{3} = \frac{{\beta \left( {\beta + 1} \right)\left( {\beta + 2} \right)\lambda^{3} }}{{\left( { - 2 + \alpha - \beta } \right)\left( { - 1 + \alpha - \beta } \right)\left( {\alpha - \beta } \right)}}$$

$$\mu^{\prime}_{4} = \frac{{\lambda^{4}\Gamma \left( { - 3 + \alpha - \beta } \right)\Gamma \left( {\beta + 4} \right)}}{{\Gamma \left( {1 + \alpha - \beta } \right)\Gamma \left( \beta \right)}}$$

The central moments about the mean are given by

$$\mu_{2} = \frac{{\alpha \beta \lambda^{2} }}{{\left( { - 1 + \alpha - \beta } \right)\left( {\alpha - \beta } \right)^{2} }},$$

$$\mu_{3} = \frac{{2\alpha \beta \left( {\alpha + \beta } \right)\lambda^{3} }}{{\left( { - 2 + \alpha - \beta } \right)\left( { - 1 + \alpha - \beta } \right)\left( {\alpha - \beta } \right)^{3} }},$$

$$\mu_{4} = \frac{{3\alpha \beta \left( { - \alpha \left( { - 2 + \beta } \right)\beta + 2\beta^{2} + \alpha^{2} \left( {2 + \beta } \right)} \right)\lambda^{4} }}{{\left( { - 3 + \alpha - \beta } \right)\left( { - 2 + \alpha - \beta } \right)\left( { - 1 + \alpha - \beta } \right)\left( {\alpha - \beta } \right)^{4} }}.$$

Hence; the mean ($\mu )$ and variance ($\sigma^{2} )$ of WLx distribution are $\mu = \frac{\beta \lambda }{\alpha - \beta }$ and $\sigma^{2} = \frac{{\alpha \beta \lambda^{2} }}{{\left( { - 1 + \alpha - \beta } \right)\left( {\alpha - \beta } \right)^{2} }}$.

The coefficients of skewness ($$\beta_{1} )$$, kurtosis ($$\beta_{2} )$$ and variation ($$CV)$$ of WLx distribution are given by:

$$\beta_{1} = \sqrt {\frac{{\left( {2\Gamma \left( {\alpha - \beta } \right)^{3}\Gamma \left( {1 + \beta } \right)^{3} - 3\Gamma \left( { - 1 + \alpha - \beta } \right)\Gamma \left( {\alpha - \beta } \right)\Gamma \left( {1 + \alpha - \beta } \right)\Gamma \left( \beta \right)\Gamma \left( {1 + \beta } \right)\Gamma \left( {2 + \beta } \right) +\Gamma \left( { - 2 + \alpha - \beta } \right)\Gamma \left( {1 + \alpha - \beta } \right)^{2}\Gamma \left( \beta \right)^{2}\Gamma \left( {3 + \beta } \right)} \right)^{2} }}{{\left( { -\Gamma \left( {\alpha - \beta } \right)^{2}\Gamma \left( {1 + \beta } \right)^{2} +\Gamma \left( { - 1 + \alpha - \beta } \right)\Gamma \left( {1 + \alpha - \beta } \right)\Gamma \left( \beta \right)\Gamma \left( {2 + \beta } \right)} \right)^{3} }}}$$

$$\beta_{2} = \frac{1}{{\left( {\Gamma \left( {\alpha - \beta } \right)^{2} {\Gamma }\left( {1 + \beta } \right)^{2} -\Gamma \left( { - 1 + \alpha - \beta } \right)\Gamma \left( {1 + \alpha - \beta } \right)\Gamma \left( \beta \right)\Gamma \left( {2 + \beta } \right)} \right)^{2} }} \times \left( {\Gamma \left( {\alpha - \beta } \right){\Gamma }\left( {1 + \beta } \right)\left[ { - 3{\Gamma }\left( {\alpha - \beta } \right)^{3}\Gamma \left( {1 + \beta } \right)^{3} + 6\Gamma \left( { - 1 + \alpha - \beta } \right)\Gamma \left( {\alpha - \beta } \right)\Gamma \left( {1 + \alpha - \beta } \right)\Gamma \left( \beta \right)\Gamma \left( {1 + \beta } \right)\Gamma \left( {2 + \beta } \right) - 4\Gamma \left( { - 2 + \alpha - \beta } \right)\Gamma \left( {1 + \alpha - \beta } \right)^{2}\Gamma \left( \beta \right)^{2}\Gamma \left( {3 + \beta } \right)} \right] +\Gamma \left( { - 3 + \alpha - \beta } \right)\Gamma \left( {1 + \alpha - \beta } \right)^{3}\Gamma \left( \beta \right)^{3}\Gamma \left( {4 + \beta } \right)} \right)$$

$$CV = \frac{{\left( {\alpha - \beta } \right)\sqrt {\frac{{\lambda^{2} \left( { -\Gamma \left( {1 + \beta } \right)^{2} + \frac{{\left( {\alpha - \beta } \right)\Gamma \left( \beta \right)\Gamma \left( {2 + \beta } \right)}}{ - 1 + \alpha - \beta }} \right)}}{{\left( {\alpha - \beta } \right)^{2} }}} }}{{\lambda\Gamma \left( {1 + \beta } \right)}}$$

Moreover, the moment generating $M_{x} \left( t \right)$ and characteristic functions $\phi_{x} \left( t \right)$ are

$$M_{x} \left( t \right) = \frac{{\frac{{1 + \lambda - t\lambda }}{{\left( { - 1 + t\lambda } \right)^{2} }} + e^{{ - t\lambda }} \left( { - t} \right)^{\alpha } \alpha \lambda ^{\alpha } \lambda \left( {1 + \lambda } \right)\Gamma \left( { - \alpha , - t\lambda } \right)}}{{\left( {1 + \lambda } \right)^{2} }},$$

$$\phi_{x} \left( t \right) = \frac{{\pi {\text{Csc}}\left( {\pi \left( {\alpha - \beta } \right)} \right)\left( {\frac{{\left( { - {\text{i}}t} \right)^{1 + \alpha - \beta } \lambda^{1 + \alpha - \beta }\Gamma \left( {1 + \alpha } \right) _{1} \tilde{F}_{1} \left( {1 + \alpha ,2 + \alpha - \beta , - {\text{i}}\lambda t} \right)}}{{\Gamma \left[ \beta \right]}} - _{1} \tilde{F}_{1} \left( {\beta , - \alpha + \beta ; - {\text{i}}\lambda t} \right)} \right)}}{{\Gamma \left( {1 + \alpha - \beta } \right)\left( {1 + \theta } \right)^{2} \left( {\theta - {\text{i}}t} \right)^{2} }},$$

where $\Gamma \left( {a,z} \right)$ is the incomplete gamma function, and ${\text{Csc}}\left( z \right)$ is the cosecant of *z*.

### Lorenz and Bonferroni curves

The Bonferroni, Lorenz curves and Gini index have many applications in economics and ecology to describe inequality distribution of wealth or size like a reliability, medicine and insurance. For WLx distribution, Lorenz and Bonferroni curves are:

$$L_{F} \left[ {F\left( x \right)} \right] = \frac{{x^{1 + \beta } \lambda^{ - 1 - \beta }\Gamma \left( {1 + \alpha } \right) _{2} \tilde{F}_{1} \left( {1 + \alpha ,1 + \beta ,2 + \beta ; - \frac{x}{\lambda }} \right)}}{{\Gamma \left( {\alpha - \beta } \right)}}$$

and                                                            

$$B_{F} \left( {F\left( x \right)} \right) = \frac{{\left( { - \alpha + \beta } \right){\text{B}}\left( { - \frac{x}{\lambda };1 + \beta , - \alpha } \right)}}{{\beta {\text{B}}\left( { - \frac{x}{\lambda };\beta , - \alpha } \right)}}$$.

The scaled total time on test transform of a distribution function is given by

$$S_{F} \left[ {F\left( t \right)} \right] = \frac{{x\left( {\alpha - \beta - \frac{{x^{\beta } \lambda^{ - \beta }\Gamma \left( {1 + \alpha } \right) _{2} \tilde{F}_{1} \left( {1 + \alpha ,\beta ,2 + \beta , - \frac{x}{\lambda }} \right)}}{{\Gamma \left( {\alpha - \beta } \right)}}} \right)}}{\beta \lambda }$$

Hence, Gini index is obtained from the relationship $G = 1 - C_{F}$; where $C_{F} = \int \nolimits_{0}^{1} S_{F} \left[ {F\left( t \right)} \right]f\left( t \right)dt$.

### Entropies

An entropy is a measure of variation or uncertainty of a random variable *X*. The theory of entropy has been successfully used in a wide diversity of applications and has been used for the characterization of numerous standard probability distributions. Two popular entropy measures are the Shannon and Rényi entropies.

The Rényi entropy of a random variable with p.d.f. *f* (*x*) is defined as

$$I_{\delta } = \frac{1}{1 - \delta }log\int \nolimits_{0}^{\infty } f^{\delta } \left( x \right)dx,\quad \delta > 0,\quad \delta \ne 1$$

For WLx distribution, Rényi entropy is derived as

$$\begin{aligned} I_{\delta } & = \frac{1}{1 - \delta }log\left( {\frac{{\Gamma \left( {\alpha + 1} \right)\lambda^{\alpha - \beta + 1} }}{{\Gamma \left( {\alpha - \beta + 1} \right)\Gamma \left( \beta \right)}}} \right)^{\delta } \int \nolimits_{0}^{\infty } \left( {\frac{{x^{\beta - 1} }}{{\left( {x + \lambda } \right)^{\alpha + 1} }}} \right)^{\delta } dx \\ & = log\lambda + \delta \left( {1 - \delta } \right)^{ - 1} log\left( {\frac{{\Gamma \left( {\alpha + 1} \right)}}{{\Gamma \left( {\alpha - \beta + 1} \right){\Gamma}\left( \beta \right)}}} \right) + \left( {1 - \gamma } \right)^{ - 1} log\left( {\frac{{\Gamma \left( { - 1 + (2 - \alpha - \beta } \right)\delta )\Gamma \left( {1 + (\beta - 1} \right)\delta }}{{\Gamma \left( {\delta + \alpha \delta } \right)}}} \right) \\ \end{aligned}$$

The Shannon entropy of a random variable *X* is defined by

$$S_{H} = - \int \nolimits_{0}^{\infty } f\left( x \right)logf\left( x \right)dx$$

Using the WLx density,

$$\begin{aligned} S_{H} & = - \frac{{\Gamma \left( {\alpha + 1} \right)\lambda^{\alpha - \beta + 1} }}{{\Gamma \left( {\alpha - \beta + 1} \right){\Gamma}\left( \beta \right)}}\int \nolimits_{0}^{\infty } \frac{{x^{\beta - 1} }}{{\left( {x + \lambda } \right)^{\alpha + 1} }}log\frac{{\Gamma \left( {\alpha + 1} \right)\lambda^{1 + \alpha - \beta } }}{{\Gamma \left( {1 + \alpha - \beta } \right)\Gamma \left( \beta \right)}}\left( {x^{\beta - 1} \left( {x + \lambda } \right)^{ - \alpha - 1} } \right)dx \\ & = - log\frac{{\Gamma \left( {\alpha + 1} \right)\lambda^{\alpha - \beta + 1} }}{{\Gamma \left( {\alpha - \beta + 1} \right){\Gamma}\left( \beta \right)}} - \left( {\beta - 1} \right)\left( {log\lambda - \psi \left( {\alpha - \beta + 1} \right) + \psi \left( \beta \right)} \right) + \left( {\alpha + 1} \right)\left( {H_{\alpha } - H_{\alpha - \beta } + log\lambda } \right) \\ & = log\frac{{\lambda\Gamma \left( {\alpha - \beta + 1} \right){\Gamma}\left( \beta \right)}}{{\Gamma \left( {\alpha + 1} \right)}} + \left( {\alpha + 1} \right)H_{\alpha } + \left( {\beta - \alpha - 2} \right)H_{\alpha - \beta } - \left( {\beta - 1} \right)\left( {\gamma + \psi \left( \beta \right)} \right) \\ \end{aligned}$$

where $H_{n}$ is the *n*-th harmonic number, $\gamma$ is the Euler–Mascheroni constant and $\psi \left( z \right)$ is the digamma function.

### Reliability parameter

In the context of reliability, the stress–strength model describes the life of a component that has a random strength *Y* that is subjected to a random stress *X*. The component fails at the instant that the stress applied to it exceeds the strength, and the component will function satisfactorily whenever $X < Y$. Hence, $R = { \Pr }(X < Y)$ is a measure of component reliability. It has many applications especially in the areas of engineering and stress–strength models. The reliability parameter for WLx distribution is given by

$$\begin{aligned} R & = \Pr \left( {X < Y} \right) \\ & = \frac{{\lambda_{1}^{{\alpha_{1} - \beta_{1} + 1}} {\Gamma}\left( {\alpha_{1} + 1} \right)}}{{\Gamma \left( {\alpha_{1} - \beta_{1} + 1} \right){\Gamma}\left( {\beta_{1} } \right)}} \times \frac{{\lambda_{2}^{{\alpha_{2} - \beta_{2} + 1}}\Gamma \left( {\alpha_{2} + 1} \right)}}{{\Gamma \left( {\alpha_{2} - \beta_{2} + 1} \right){\Gamma}\left( {\beta_{2} } \right)}} \times \int \nolimits_{0}^{\infty } \int \nolimits_{0}^{y} \left( {\frac{{x^{{\beta_{1} - 1}} }}{{\left( {x + \lambda_{1} } \right)^{{\alpha_{1} + 1}} }}} \right)\left( {\frac{{y^{{\beta_{2} - 1}} }}{{\left( {y + \lambda_{2} } \right)^{{\alpha_{2} + 1}} }}} \right)dxdy \\ & = \frac{{\lambda_{1}^{{ - \beta_{1} }}\Gamma \left( {\alpha_{1} + 1} \right)}}{{\Gamma \left( {\alpha_{1} - \beta_{1} + 1} \right)}} \times \frac{{\lambda_{2}^{{\alpha_{2} - \beta_{2} + 1}} {\Gamma}\left( {\alpha_{2} + 1} \right)}}{{\Gamma \left( {\alpha_{2} - \beta_{2} + 1} \right)\Gamma \left( {\beta_{2} } \right)}} \times \int \nolimits_{0}^{\infty } \frac{{y^{{\beta_{1} + \beta_{2} - 1}} }}{{\left( {y + \lambda_{2} } \right)^{{\alpha_{2} + 1}} }} _{2} \hat{F}_{1} \left(\alpha_{1} + 1,\beta_{1} ,\beta_{1} + 1; - \frac{y}{{\lambda_{1} }}\right)dy \\ & = \left\{ {\left[ {\frac{{\left( {\lambda_{2} /\lambda_{1} } \right)^{{\alpha_{2} - \beta_{2} + 1}}\Gamma \left( {\alpha_{2} + 1} \right){\Gamma}\left( {2 + \alpha_{1} + \alpha_{2} - \beta_{1} - \beta_{2} } \right)\Gamma \left( { - 1 - \alpha_{2} + \beta_{1} + \beta_{2} } \right)}}{{\left( {\alpha_{2} - \beta_{2} + 1} \right)\Gamma \left( {\beta_{1} } \right){\Gamma}\left( {\beta_{2} } \right)\left( {\alpha_{1} - \beta_{1} + 1} \right)\Gamma \left( {\alpha_{2} - \beta_{2} + 1} \right)}}} \right.} \right. \\ & \quad\times \left. { _{p} F_{q} \left( {\left\{ {1 + \alpha_{2} ,1 + \alpha_{2} - \beta_{2} ,2 + \alpha_{1} + \alpha_{2} - \beta_{1} - \beta_{2} } \right\},\left\{ {2 + \alpha_{2} - \beta_{2} ,2 + \alpha_{2} - \beta_{1} - \beta_{2} } \right\};\frac{{\lambda_{2} }}{{\lambda_{1} }}} \right)} \right] \\ & \quad+ \left[ {\frac{{\left( {\lambda_{2} /\lambda_{1} } \right)^{{\beta_{1} }}\Gamma \left( {\alpha_{1} + 1} \right){\Gamma}\left( {1 + \alpha_{2} - \beta_{1} - \beta_{2} } \right)\Gamma \left( {\beta_{1} + \beta_{2} } \right)}}{{\left( {\alpha_{2} - \beta_{2} + 1} \right)\Gamma \left( {\beta_{1} } \right){\Gamma}\left( {\beta_{2} } \right)\left( {\alpha_{1} - \beta_{1} + 1} \right)\Gamma \left( {\alpha_{2} - \beta_{2} + 1} \right)}}} \right.\\ &\quad\times \left. {\left. { _{p} F_{q} \left( {\left\{ {1 + \alpha_{1} ,\beta_{1} ,\beta_{1} + \beta_{2} } \right\},\left\{ {1 + \beta_{1} , - \alpha_{2} + \beta_{1} + \beta_{2} } \right\};\frac{{\lambda_{2} }}{{\lambda_{1} }}} \right) } \right]} \right\} \\ \end{aligned}$$

where $_{p} F_{q}$($\left\{ {a_{1} ,a_{2} , \ldots ,a_{p} } \right\},\left\{ {b_{1} ,b_{2} , \ldots ,b_{1} } \right\};z$) is the generalized hypergeometric function.

## Parameter estimation

In this section, we consider the method of moments and the maximum likelihood techniques is considered to estimate the involved parameters of WLx distribution. In addition, the interval estimation is discussed via Fisher information matrix.

### Method of moments estimates

The method of moment estimation (MME) consists of equating the first three moments of the population (7), (8) and (9) to the corresponding moments of the sample. The system of equations that needed is:

10 $$\frac{\beta \lambda }{\alpha - \beta } = m_{1}$$

11 $$\frac{{\beta \left( {\beta + 1} \right)\lambda^{2} }}{{\left( { - 1 + \alpha - \beta } \right)\left( {\alpha - \beta } \right)}} = m_{2}$$

12 $$\frac{{\beta \left( {\beta + 1} \right)\left( {\beta + 2} \right)\lambda^{3} }}{{\left( { - 2 + \alpha - \beta } \right)\left( { - 1 + \alpha - \beta } \right)\left( {\alpha - \beta } \right)}} = m_{3}$$

By solving the Eqs. (10), (11) and (12), the parameter estimates are

$$\begin{aligned} \hat{\lambda } & = \frac{{m_{1} m_{2}^{2} - 2m_{1}^{2} m_{3} + m_{2} m_{3} }}{{\left( {m_{1}^{2} - 2m_{2} } \right)m_{2} + m_{1} m_{3} }} \\ \hat{\alpha } & = \frac{{2\left( {m_{1}^{2} - m_{2} } \right)\left( {m_{1} m_{2} - m_{3} } \right)\left( {m_{2}^{2} - m_{1} m_{3} } \right)}}{{\left( {\left( {m_{1}^{2} - 2m_{2} } \right)m_{2} + m_{1} m_{3} } \right)\left( { - m_{1} m_{2}^{2} + \left( {2m_{1}^{2} - m_{2} } \right)m_{3} } \right)}} \\ \hat{\beta } & = \frac{{2m_{1} \left( {m_{2}^{2} - m_{1} m_{3} } \right)}}{{ - m_{1} m_{2}^{2} + \left( {2m_{1}^{2} - m_{2} } \right)m_{3} }}. \\ \end{aligned}$$

### Maximum likelihood estimates

Let $x_{1} ,x_{2} , \ldots ,x_{n}$ be a random sample of size n from the WLx distribution, the maximum likelihood estimates (MLEs) of the parameters are obtained by direct maximization of the log-likelihood function which is given by:

$$\begin{aligned} \ln \left( {L\left( {x;\lambda ,\alpha ,\beta } \right)} \right) & = n ln\left[ {\Gamma \left( {\alpha + 1} \right)} \right] + n\left( {\alpha - \beta + 1} \right) ln\left[ \lambda \right] - n ln\left[ {\Gamma \left( {\alpha - \beta + 1} \right)} \right] \\ & \quad - n ln\left[ {\Gamma \left( \beta \right)} \right] + \left( {\beta - 1} \right)\mathop \sum \limits_{i = 1}^{n} \ln \left[ {x_{i} } \right] - \left( {\alpha + 1} \right)\mathop \sum \limits_{i = 1}^{n} ln\left[ {x_{i} + \lambda } \right] \\ \end{aligned}$$

It follows that the maximum likelihood estimators (MLEs); $\hat{\lambda }$, $\hat{\alpha }$ and $\hat{\beta }$ are the simultaneous solutions of the equations:

$$\frac{{n\left( {1 + \alpha - \beta } \right)}}{\lambda } - \left( {1 + \alpha } \right)\mathop \sum \limits_{i = 1}^{n} \frac{1}{{x_{i} + \lambda }} = 0$$

$$nln\left[ \lambda \right] + n\psi \left( {\alpha + 1} \right) - n\psi \left( {\alpha - \beta + 1} \right) - \mathop \sum \limits_{i = 1}^{n} ln\left[ {\lambda + x_{i} } \right] = 0$$

$$- nln\left[ \lambda \right] + n\psi \left( {\alpha - \beta + 1} \right) - n\psi \left( \beta \right) + \mathop \sum \limits_{i = 1}^{n} ln\left[ {x_{i} } \right] = 0$$

For interval estimation of the parameter vector $\varTheta = \left( {\lambda ,\alpha , \beta } \right)^{T}$; the expected fisher information matrix ${\mathbf{I}} = \left[ {I_{ij} } \right], i,j = 1,2,3$ is derived as follows:

$$\begin{aligned} I_{11} &= - E\left[ {\frac{{\partial^{2} }}{{\partial \lambda^{2} }}\ln f\left( x \right)} \right] = \frac{{\pi {\text{Csc}}\left( {\pi \left( {\alpha - \beta } \right)} \right)\left( {\frac{{\left( {1 + \alpha - \beta } \right)^{2} \left( {2 + \alpha - \beta } \right)\left( {\frac{1 + \alpha }{\beta }} \right)^{ - \alpha } \left( {\frac{ - 1 - \alpha + \beta }{\beta }} \right)^{\alpha - \beta }\Gamma \left( {1 + \alpha } \right)}}{{\left( {1 + \alpha } \right)\Gamma \left( \beta \right)}} + \frac{{ - 2 + _{2} F_{1} \left[ {1,\beta , - 1 - \alpha + \beta ;\frac{ - 1 - \alpha + \beta }{\beta }} \right]}}{{\Gamma \left( { - 2 - \alpha + \beta } \right)}}} \right)}}{{\lambda\Gamma \left( {3 + \alpha - \beta } \right)}};\\I_{22} &= - E\left[ {\frac{{\partial^{2} }}{{\partial \alpha^{2} }}\ln f\left( x \right)} \right] = - \psi^{\prime}\left( {\alpha + 1} \right) + \psi^{\prime}\left( {\alpha - \beta + 1} \right);\\I_{33} &= - E\left[ {\frac{{\partial^{2} }}{{\partial \beta^{2} }}\ln f\left( x \right)} \right] = \psi^{\prime}\left( {\alpha - \beta + 1} \right) + \psi^{\prime}\left( \beta \right);\\I_{12} &= - E\left[ {\frac{{\partial^{2} }}{\partial \lambda \partial \alpha }\ln f\left( x \right)} \right] = - \frac{\beta }{{\lambda \left( {\alpha + 1} \right)}};\\I_{13} &= - E\left[ {\frac{{\partial^{2} }}{\partial \lambda \partial \beta }\ln f\left( x \right)} \right] = \frac{1}{\lambda };\\I_{23} &= - E\left[ {\frac{{\partial^{2} }}{\partial \alpha \partial \beta }\ln f\left( x \right)} \right] = - \psi^{\prime}\left( {\alpha - \beta + 1} \right); \end{aligned}$$

where $\psi^{\prime}\left( z \right)$ is the trigamma function.

Under regularity conditions, Bahadur (1964) showed that as $n \to \infty$, $\sqrt n \left( {{\hat{\varTheta }} -\Theta } \right)$ is asymptotically normal 3-variate with (vector) mean zero and covariance matrix ${\mathbf{I}}^{ - 1}$. The asymptotic variances and covariance of the elements of ${\hat{\varTheta }}$ are given by:

$$V\left( {\hat{\alpha }} \right) = \frac{{I_{22 } I_{33} - I_{23}^{2} }}{{n {\Delta}}},\quad V\left( {\hat{\beta }} \right) = \frac{{I_{11 } I_{33} - I_{13}^{2} }}{{n {\Delta}}},\quad V\left( {\hat{\theta }} \right) = \frac{{I_{11 } I_{22} - I_{12}^{2} }}{{n {\Delta}}},\quad Cov\left( {\hat{\alpha },\hat{\beta }} \right) = \frac{{I_{13 } I_{23} - I_{12 } I_{33} }}{{n {\Delta}}},\quad Cov\left( {\hat{\alpha },\hat{\theta }} \right) = \frac{{I_{12 } I_{23} - I_{13 } I_{22} }}{{n {\Delta}}},\quad Cov\left( {\hat{\beta },\hat{\theta }} \right) = \frac{{I_{13 } I_{12} - I_{11 } I_{23} }}{{n {\Delta}}}.$$

where $\Delta = \det \left( {\mathbf{I}} \right)$. The corresponding asymptotic $100\left( {1 - \alpha } \right){\text{\% }}$ confidence intervals are ${\hat{\Theta }} \pm c {\mathbf{I}}^{ - 1/2}$; where c is the appropriate *z* critical value.

## Order statistics and extreme values

The distribution of extreme values plays an important role in statistical applications. In this section the probability and cumulative function of order statistics are introduced and the limiting distribution minimum and the maximum arising from the WLx model can be derived.

### Probability and cumulative function of order statistics

Suppose *X*_1_ *, X*_2_ *, …, X*_*n*_ is a random sample from WLx distribution. Let *X*_1:*n*_ < *X*_2:*n*_ < · · · < *X*_*n*:*n*_ denote the corresponding order statistics. The probability density function and the cumulative distribution function of the *j*th order statistic, say *Y* = *X*_*j*:*n*_, are given by

$$\begin{aligned} f_{Y} \left( y \right) & = \frac{n!}{{\left( {j - 1} \right)!\left( {n - j} \right)!}}F^{j - 1} \left( y \right)\{ 1 - F\left( y \right)\}^{n - j} f\left( y \right) \\ & = \frac{{\lambda^{1 + \alpha } \left( {y + \lambda } \right)^{ - 1 - \alpha } n!}}{{y\Gamma \left( j \right){\Gamma}\left( {1 - j + n} \right)\Gamma \left( \beta \right) _{2} \tilde{F}_{1} \left( {1 + \alpha ,\beta ,1 + \beta ; - \frac{y}{\lambda }} \right)}} \left[ {\frac{{y^{\beta } \lambda^{ - \beta }\Gamma \left( {1 + \alpha } \right) _{2} \tilde{F}_{1} \left( {1 + \alpha ,\beta ,1 + \beta ; - \frac{y}{\lambda }} \right)}}{{\Gamma \left( {1 + \alpha - \beta } \right)}}} \right]^{j} \\ & \times \left[ {1 - \frac{{y^{\beta } \lambda^{ - \beta } {\Gamma}\left( {1 + \alpha } \right) _{2} \tilde{F}_{1} \left( {1 + \alpha ,\beta ,1 + \beta ; - \frac{y}{\lambda }} \right)}}{{\Gamma \left( {1 + \alpha - \beta } \right)}}} \right]^{n - j} \\ \end{aligned}$$

and

$$\begin{aligned} F_{Y} \left( y \right) & = \mathop \sum \limits_{m = j}^{n} \left( {\begin{array}{*{20}c} n \\ m \\ \end{array} } \right){\text{F}}^{m} \left( y \right) \times \left[ {1 - {\text{F}}\left( {\text{y}} \right)} \right]^{n - m} \\ & = \frac{{\Gamma \left( {1 + n} \right) _{2} \tilde{F}_{1} \left( {1,j - n, + j;\frac{1}{{1 - \frac{{y^{ - \beta } \lambda^{\beta }\Gamma \left( {1 + \alpha - \beta } \right)}}{{\Gamma \left( {1 + \alpha } \right) _{2} \tilde{F}_{1} \left( {1 + \alpha ,\beta ,1 + \beta ; - \frac{y}{\lambda }} \right)}}}}} \right)}}{{\Gamma \left( {1 - j + n} \right)}} \\ \quad\times \left[ {\frac{{y^{\beta } \lambda^{ - \beta } {\Gamma}\left( {1 + \alpha } \right) _{2} \tilde{F}\left( {1 + \alpha ,\beta ,1 + \beta ; - \frac{y}{\lambda }} \right)}}{{\Gamma \left( {1 + \alpha - \beta } \right)}}} \right]^{j} \left[ {1 - \frac{{y^{\beta } \lambda^{ - \beta } {\Gamma}\left( {1 + \alpha } \right) _{2} \tilde{F}\left( {1 + \alpha ,\beta ,1 + \beta , - \frac{y}{\lambda }} \right)}}{{\Gamma \left( {1 + \alpha - \beta } \right)}}} \right]^{n - j} \\ \end{aligned}$$

### Limiting distributions of extreme values

Let $m_{n} = X_{1:n} = { \hbox{min} }\left[ {X_{1} , {\text{X}}_{2} , \ldots ,{\text{X}}_{n} } \right]$ and $M_{n} = X_{n:n} = { \hbox{max} }\left[ {X_{1} , {\text{X}}_{2} , \ldots ,{\text{X}}_{n} } \right]$ arising from WLx distribution. The limiting distributions of $X_{1:n}$ and $X_{n:n}$ can be derived in the following theorem.

#### **Theorem 3**

*Let* $m_{n}$ *and* $M_{n}$ *be the minimum and the maximum of a random sample from the WLx distribution, respectively. Then*

(i) $$\mathop {\lim }\nolimits_{n \to \infty } p\left( {\frac{{m_{n} - a_{n} }}{{b_{n} }} \le x} \right) = 1 - { \exp }\left( { - x^{\beta } } \right);\quad x > 0$$
(ii) $$\mathop {\lim }\nolimits_{n \to \infty } p\left( {\frac{{M_{n} - c_{n} }}{{d_{n} }} \le x} \right) = { \exp }\left( { - x^{{ - \left( {1 + \alpha - \beta } \right)}} } \right);\quad x > 0$$
*where* $$a_{n} = 0$$, $$b_{n} = \frac{1}{{F^{ - 1} \left( {\frac{1}{n}} \right)}}, c_{n} = 0$$ *and* $$d_{n} = \frac{1}{{F^{ - 1} \left( {1 - \frac{1}{n}} \right)}}$$.

#### *Proof*

(i) Using L’Hospital rule, we have

$$\mathop {\lim }\limits_{{\varepsilon \to 0^{ + } }} \frac{{F\left( {F^{ - 1} \left( 0 \right) + \varepsilon x} \right)}}{{F\left( {F^{ - 1} \left( 0 \right) + \varepsilon } \right)}} = \mathop {\lim }\limits_{{\varepsilon \to 0^{ + } }} \frac{{F\left( {\varepsilon x} \right)}}{F\left( \varepsilon \right)} = \mathop {\lim }\limits_{{\varepsilon \to 0^{ + } }} \frac{{xf\left( {\varepsilon x} \right)}}{f\left( \varepsilon \right)} = x^{\beta } .$$

Therefore by Theorem (8.3.6) of Arnold et al. (1992), the minimal domain of attraction of the WLx distribution is the Weibull distribution, proving part (i).

(ii) Using L’Hospital rule, we have

$$\mathop {\lim }\limits_{t \to \infty } \frac{{1 - F\left( {tx} \right)}}{1 - F\left( t \right)} = \mathop {\lim }\limits_{t \to \infty } \frac{{xf\left( {tx} \right)}}{f\left( t \right)} = x^{{ - \left( {1 + \alpha - \beta } \right)}}$$

Therefore, by Theorem (1.6.2) and Corollary (1.6.3) in Leadbetter et al. (1987), the maximal domain of attraction of the WLx distribution is the Fréchet distribution, proving part (ii).

## Simulation study

The equation $F\left( x \right) - u = 0$, where *u* is an observation from the uniform distribution (0,1) and $F\left( x \right)$ is cumulative distribution function of WLx distribution, is used to carry out the simulation study by generating random samples follow WLx distribution. The simulation experiment was repeated $1000$ times each with sample sizes; $20, 40, 70, 100$ for $\left( {\lambda ,\alpha ,\beta } \right) = \left( {0.03, 2, 0.5} \right)$ and $\left( {0.01, 2.5, 1} \right)$. The following measures are computed:

(i) Average bias of $\hat{\lambda }$, $\hat{\alpha }$ and $\hat{\beta }$ of the parameters $\lambda ,\alpha$ and $\beta$ are respectively;
  $$\frac{1}{N}\mathop \sum \limits_{i = 1}^{N} (\hat{\lambda } - \lambda ),\quad \frac{1}{N}\mathop \sum \limits_{i = 1}^{N} (\hat{\alpha } - \alpha ) \quad and \quad \frac{1}{N}\mathop \sum \limits_{i = 1}^{N} (\hat{\beta } - \beta )$$
(ii) The Mean square error (MSE) of $\hat{\lambda }$, $\hat{\alpha }$ and $\hat{\beta }$ of the parameters $\lambda ,\alpha$ and $\beta$ are respectively;
  $$\frac{1}{N}\mathop \sum \limits_{i = 1}^{N} \left( {\hat{\lambda } - \lambda } \right)^{2} ,\quad \frac{1}{N}\mathop \sum \limits_{i = 1}^{N} \left( {\hat{\alpha } - \alpha } \right)^{2} \quad and \quad \frac{1}{N}\mathop \sum \limits_{i = 1}^{N} \left( {\hat{\beta } - \beta } \right)^{2}$$

Table 1 presents the average bias and the MSE of the estimates. The values of the bias are seen to be small,positive and the values of the MSEs decreases while the sample size increases.

**Table 1:** Bias and MSE for the parameters $\lambda ,\alpha$, $\beta$

$\lambda$	*α*	*β*	n	Bias (*λ*)	MSE (*λ*)	Bias (*α*)	MSE (*α*)	Bias (*β*)	MSE (*β*)
0.03	2	0.5	20	−0.0285	0.00299	4.6345	35.5596	4.7126	36.5100
			40	−0.0266	0.00071	3.8839	23.5742	4.0339	24.7811
			70	−0.0261	0.00068	3.1262	15.2725	3.3071	16.3899
			100	−0.0257	0.00066	2.7709	11.9247	2.9629	12.9699
0.01	2.5	1	20	−0.0083	0.000069	5.8120	60.4177	5.8464	61.0917
			40	−0.0081	0.000066	5.1198	45.5128	5.2032	46.4064
			70	−0.0078	0.000061	4.0102	28.6942	4.1131	29.5265
			100	−0.0075	0.000058	3.4679	22.5719	3.5759	23.317

## Application

The considered a dataset corresponding to remission times (in months) of a random sample of 128 bladder cancer patients given in Lee and Wang (2003). The data are given as follows: 0.08, 2.09, 3.48, 4.87, 6.94, 8.66, 13.11, 23.63, 0.20, 2.23, 3.52, 4.98, 6.97, 9.02, 13.29, 0.40, 2.26, 3.57, 5.06, 7.09, 9.22, 13.80, 25.74, 0.50, 2.46, 3.64, 5.09, 7.26, 9.47, 14.24, 25.82, 0.51, 2.54, 3.70, 5.17, 7.28, 9.74, 14.76, 26.31, 0.81, 2.62, 3.82, 5.32, 7.32, 10.06, 14.77, 32.15, 2.64, 3.88, 5.32, 7.39, 10.34, 14.83, 34.26, 0.90, 2.69, 4.18, 5.34, 7.59, 10.66, 15.96, 36.66, 1.05, 2.69, 4.23, 5.41, 7.62, 10.75, 16.62, 43.01, 1.19, 2.75, 4.26, 5.41, 7.63, 17.12, 46.12, 1.26, 2.83, 4.33, 5.49, 7.66, 11.25, 17.14, 79.05, 1.35, 2.87, 5.62, 7.87, 11.64, 17.36, 1.40, 3.02, 4.34, 5.71, 7.93, 11.79, 18.10, 1.46, 4.40, 5.85, 8.26, 11.98, 19.13, 1.76, 3.25, 4.50, 6.25, 8.37, 12.02, 2.02, 3.31, 4.51, 6.54, 8.53, 12.03, 20.28, 2.02, 3.36, 6.76, 12.07, 21.73, 2.07, 3.36, 6.93, 8.65, 12.63, 22.69. We have fitted the WLx distribution to the dataset using MLE, and compared the proposed WLx distribution with, gamma-Lomax (GL), Kumaraswamy-Lomax(KwL), transmuted exponentiated-Lomax (TrEL), Weibull-Lomax (WL), McDonald-Lomax (McL), Beta-Lomax(BL), extended Poisson-Lomax (EPL), exponential-Lomax (ExL) and Lomax distributions.

The c.d.f.(s) of these models are given as follow:

- The McLomax (McL) density function with five parameters $\alpha ,\lambda , \beta , a$ and *b* introduced by Lemonte and Cordeiro (2013) is expressed as
  $$f_{McL} \left( x \right) = \frac{{ \beta \alpha \lambda^{\alpha } \left( {\lambda + x} \right)^{{ - \left( {\alpha + 1} \right)}} }}{{B\left( {a \beta^{ - 1} ,b + 1} \right)}}\left( {1 - \left( {\frac{\lambda }{\lambda + x}} \right)^{\alpha } } \right)^{a - 1} \left( {1 - \left( {1 - \left( {\frac{\lambda }{\lambda + x}} \right)^{\alpha } } \right)^{ \beta } } \right)^{b} ,$$
  (where $$x > 0 ;\alpha ,\lambda ,\beta ,b,a, > 0$$).
  Evidently, the McL density function does not involve any complicated function, and it includes several distributions as special sub-models not previously considered in the literature. In fact, the Lomax distribution (with parameters $\alpha$ and $\lambda$) is clearly a basic exemplar for $a = \beta = 1$ and $b = 0$. Beta Lomax (BL) and Kumaraswamy Lomax (KwL) distributions are new models which arise for $\beta = 1$ and $a = \beta$, respectively. For $b = 0$ and $\beta = 1$, it leads to a new distribution referred to as the Exponentiated Lomax (EL) distribution. The McL distribution allows for greater flexibility of its tails and can be widely applied in many areas. The c.d.f. corresponding to McL density function is given by
  $$F_{McL} \left( x \right) = I_{{\left\{ {1 - \lambda^{\alpha } \left( {\lambda + x} \right)^{ - \alpha } } \right\}^{\beta } }} \left( {a\beta^{ - 1} ,b + 1} \right)$$
- The Exponential Lomax (ExL) distribution introduced by El-Bassiouny et al. (2015) with c.d.f.
  $$F_{EL} \left( x \right) = 1 - {\text{e}}^{{ - \beta \times \left( {\frac{\lambda }{x + \lambda }} \right)^{ - \alpha } }} , \quad x \ge - \lambda ,\quad \alpha ,\quad \lambda ,\quad \beta > 0$$
- The gamma-Lomax (GL) distribution introduced by Cordeiro et al. (2015) based on a versatile and flexible gamma generator proposed by Zagrafos and Balakrishnan (2009) using Stacy’s generalized gamma distribution and record value theory. The c.d.f. of GL distribution is given by
  $$F_{GL} \left( x \right) = \frac{{\Gamma \left[ {a,\upalpha{\text{Log}}\left[ {1 + \frac{x}{\lambda }} \right]} \right]}}{{\Gamma \left[ a \right]}},\quad x > 0,\quad \alpha ,\lambda ,a > 0$$
- The transmuted Exponentiated-Lomax (TrEL) distribution introduced by Ashour and Eltehiwy (2013) with c.d.f.
  $$F\left( x \right) = \left( {1 - \left( {1 + \lambda x} \right)^{ - \alpha } } \right)^{\beta } \left( {\left( {1 + \gamma } \right) - \gamma \left( {1 - \left( {1 + \lambda x} \right)^{ - \alpha } } \right)^{\beta } } \right),$$
  (where $$> 0;\alpha ,\lambda ,\beta ,\gamma > 0$$)
- The Weibull–Lomax (WL) distribution introduced by Tahir et al. (2015) with c.d.f.
  $$F_{WL} \left( x \right) = 1 - {\text{e}}^{{\left( { - a\left( {\left( {1 + \left( {\frac{x}{\lambda }} \right)} \right)^{\alpha } - 1} \right)^{b} } \right)}} ,\quad x > 0,\quad a,b,\alpha ,\lambda > 0$$
- The extended Poisson-Lomax (EPL) distribution introduced by Al-Zahrani et al. (2015) introduced with c.d.f.
  $$F_{EPL} \left( x \right) = 1 - \left( {1 + \lambda x} \right)^{ - \alpha } {\text{e}}^{{ - \beta \left( {1 - \left( {1 + \lambda x} \right)^{ - \alpha } } \right)}} ,\quad x > 0;\beta \ge 0,\alpha ,\lambda > 0$$

The model selection is carried out using the Akaike information criterion (AIC), the Bayesian information criterion (BIC), the Hannan-Quinn information criterion (HQIC) and the consistent Akaike information criteria (CAIC) defined by:

$${\text{AIC}} = - 2l\left( {\hat{\varvec{\theta }}} \right) + 2q$$

$${\text{BIC}} = - 2l\left( {\hat{\varvec{\theta }}} \right) + qlog\left( n \right)$$

$${\text{HQIC}} = - 2l\left( {\hat{\varvec{\theta }}} \right) + 2qlog\left( {log\left( n \right)} \right)$$

$${\text{CAIC}} = - 2l\left( {\hat{\varvec{\theta }}} \right) + \frac{2qn}{n - q - 1}$$

where $l\left( {\hat{\varvec{\theta }}} \right)$ denotes the log-likelihood function evaluated at the maximum likelihood estimates for parameters $\varvec{\theta}$, *q* is the number of parameters, and *n* is the sample size.

Table 2 provide the MLEs of the model parameters. The model with minimum AIC (or BIC, CAICand HQIC) value is chosen as the best model to fit the data. From Table 3, we note that the WLx model gives the lowest values for the AIC, BIC, HQIC and CAIC statistics among all fitted models. So, the WLx model could be chosen as the best model comparable GL, KwL, TrEL, WL, McL, BL, EPL, ExL and Lomax distributions.

**Table 2:** MLEs for bladder cancer data

Distribution	MLEs					
	$\hat{\lambda }$	$\hat{\alpha }$	$\hat{\beta }$	${\hat{\gamma }}$	$\hat{a}$	$\hat{b}$
1. WLx	20.8789	5.1265	1.5857	–	–	–
2. GL	20.5807	4.7541	–	–	1.5858	–
3. KwL	12.2973	0.3911	–	–	1.5162	11.0323
4. TrEL	0.0546	3.3391	1.71418	0.2440	–	–
5. WL	1.5794	0.2566	–	–	2.4215	1.8639
6. McL	11.2929	0.8085	2.1046	–	1.5060	4.1886
7. BL	23.9281	3.9191	–	–	1.5853	0.1572
8. Lomax	121.0225	13.9384	–	–	–	–
9. EPL	0.00804	0.2387	59.8378	–	–	–
10. ExL	0.0800	1.0644	0.0060	–	–	–

**Table 3:** The Measures AIC, BIC, HQIC, CAIC for bladder cancer data

Distribution	Measures				
	−Log L	AIC	BIC	HQIC	CAIC
1. WLx	−410.07	826.14	834.70	829.62	826.33
2. GL	−410.08	826.16	834.71	829.64	826.36
3. KwL	−409.94	827.88	839.29	832.52	828.14
4. Tr EL	−410.43	828.87	840.2	833.51	829.13
5. WL	−410.81	829.62	841.03	834.26	829.88
6. McL	−409.91	829.82	844.09	835.62	830.14
7. BL	−411.74	831.47	842.89	836.12	831.74
8. Lomax	−413.83	831.67	837.37	833.98	831.80
9. EPL	−413.83	833.67	842.22	837.14	833.86
10. ExL	−414.98	835.96	844.51	839.43	836.15

Now, the formal goodness-of-fit tests are applied in order to verify which distribution fits better to these data. The Cramér–von Mises ($W_{n}^{2}$), Anderson–Darling ($A_{n}^{2}$), Watson ($U_{n}^{2}$) and Liao-Shimokawa ($L_{n}$) tests statistics are considered. For further details, the reader is refereed to Chen and Balakrishnan (1995). In general, the smaller the values of the $W_{n}^{2}$, $A_{n}^{2}$, $U_{n}^{2}$ and $L_{n}$, the better the fit to the data. The values of the statistics $W_{n}^{2}$, $A_{n}^{2}$, $U_{n}^{2}$ and $L_{n}$ are given in Table 4. Based on these statistics, the WLx model fits the bladder cancer data better than TrEL, WL, EPL, EXL and Lomax models and gives values close to GL and BL it can be concluded.

**Table 4:** Goodness-of-fit tests for bladder cancer data

Distribution	Statistics			
	$W_{n}^{2}$	$A_{n}^{2}$	$U_{n}^{2}$	$L_{n}$
1. WLx	0.026524	0.18137	31.5282	0.47912
2. EL	0.026820	0.18341	31.5288	0.48320
3. GL	0.026190	0.18089	31.5298	0.47768
4. TrEL	0.031438	0.22753	31.5314	0.53413
5. WL	0.038295	0.26273	31.5434	0.57746
6. BL	0.025822	0.17872	31.5272	0.47508
7. Lomax	0.212589	1.37456	31.7017	1.05935
8. EPL	0.226761	1.45110	31.7139	1.07341
9. ExL	0.179676	1.0908	31.6934	1.08401

## Conclusion

In this paper, WLx distribution is proposed. A mathematical treatment of the proposed distribution including explicit formulas for the density and hazard functions, moments, order statistics have been provided. The estimation of the parameters has been approached by maximum likelihood and method of moments and the observed information matrix is obtained. The usefulness of the new distribution is illustrated in an analysis of Bladder cancer data. The results indicate that the WLx distribution applicable and more flexible than other extensions of Lomax distribution.

## References

[CR1] Abdul-Moniem IB, Abdel-Hameed HF (2012). On exponentiated Lomax distribution. Int J Math Arch.

[CR2] Afify AZ, Nofal ZM, Yousof HM, Gebaly YM, Butt NS (2015). The transmuted Weibull Lomax distribution: properties and application. Pak J Stat Oper Res.

[CR3] Al-Zahrani B (2015). An extended Poisson–Lomax distribution. Adv Math Sci J.

[CR4] Arnold BC, Balakrishnan N, Nagaraja HN (1992). A first course in order statistics.

[CR5] Ashour SK, Eltehiwy MA (2013). Transmuted exponentiated lomax distribution. Aust J Basic Appl Sci.

[CR6] Bahadur RR (1964). On Fisher’s bound for asymptotic variances. Ann Math Stat.

[CR7] Bryson MC, Siddique MM (1969). Some criteria for aging. J Am Stat Assoc.

[CR8] Chen G, Balakrishnan N (1995). A general purpose approximate goodness-of-fit test. J Qual Technol.

[CR9] Cordeiro GM, Ortega EMM, Popović BV (2015). The gamma-Lomax Distribution. J Stat Comput Simul.

[CR10] El-Bassiouny AH, Abdo NF, Shahen HS (2015). Exponential Lomax distribution. Int J Comput Appl.

[CR11] Ghitany ME, AL-Awadhi FA, Alkhalfan LA (2007). Marshall–Olkin extended Lomax distribution and its applications to censored data. Commun Stat Theory Methods.

[CR12] Glaser RE (1980). Bathtub and related failure rate characterizations. J Am Stat Assoc.

[CR13] Gupta RC, Akman O (1995). Mean residual life function for certain types of non-monotonic ageing. Commun Stat Stoch Models.

[CR14] Gupta PL, Gupta RC (1983). On the moments of residual life in reliability and some characterization results. Commun Stat Theory Methods.

[CR15] Gupta AK, Tripathi RC (1996). Weighted bivariate logarithmic series distributions. Commun Stat Theory Methods.

[CR16] Gupta RC, Ghitany ME, Al-Mutairi DK (2010). Estimation of reliability from Marshall–Olkin extended Lomax distributions. J Stat Comput Simul.

[CR17] Hewa AP (2011) Statistical properties of weighted generalized gamma distribution. Master dissertation, Statesboro, Georgia

[CR18] Kundu C, Nanda AK (2010). Some reliability properties of the inactivity time. Commun Stat Theory Methods.

[CR19] Leadbetter MR, Lindgren G, Rootzén H (1987). Extremes and related properties of random sequences and processes.

[CR20] Lee ET, Wang JW (2003). Statistical methods for survival data analysis.

[CR21] Lemonte AJ, Cordeiro GM (2013). An extended Lomax distribution. Statistics.

[CR22] Lomax KS (1987). Business failures: another example of the analysis of failure data. J Am Stat Assoc.

[CR23] Nanda AK, Singh H, Misra N, Paul P (2003). Reliability properties of reversed residual lifetime. Commun Stat Theory Methods.

[CR24] Nofal ZM, Afify AZ, Yousof HM, Cordeiro, GM (2016) The generalized transmuted-G family of distributions. Commun Stat Theory Methods (accepted)

[CR25] Oluyede BO, George EO (2002). On stochastic inequalities and comparisons of reliability measures for weighted distributions. Math Probl Eng.

[CR26] Patil GP, Ord JK (1976). On size biased sampling and related form invariant weighted distribution. Indian J Stat.

[CR27] Patil GP, Rao GR (1978). Weighted distributions and size biased sampling with applications to wildlife populations and human families. Biometrics.

[CR28] Rao CR (1965) On discrete distributions arising out of methods of a ascertainment. In: Patil GP (ed) Classical and contagious discrete distributions. Pergamon Press and Statistical Publishing Society, Calcutta, pp 320–332

[CR29] Rao CR (1985) Weighted distributions arising out of methods of ascertainment. In: Atkinson AC, Fienberg SE (eds) A celebration of statistics. Springer, New York, pp 543–569

[CR30] Stene J (1981). Probability distributions arising from the ascertainment and the analysis of data on human families and other groups. Stat Distrib Sci Work Appl Phys Soc Life Sci.

[CR31] Tahir MH, Cordeiro GM, Mansoor M, Zubair M (2015) The Weibull Lomax distribution: properties and applications. Hacettepe J Math Stat 44(2):461–480

[CR32] Zelen M, Proschan F, Sering RJ (1974). Problems in cell kinetics and the early detection of disease. Reliability and biometry.

[CR33] Zografos K, Balakrishnan N (2009). On families of beta- and generalized gamma generated distributions and associated inference. Stat Methodol.

